# Fecal Short-Chain Fatty Acids (SCFAs) and Their Role in Metabolic Disorders: A Systematic Review

**DOI:** 10.7759/cureus.91579

**Published:** 2025-09-04

**Authors:** Salman Ahmad, Murhaf Assaf, Binu Thomas, Hassan Ibrahim, Gowthami Muppuri, Prem Chand, Albina Mercy, Manesh Kumar, Rameet Kumar, Ahmed Khan

**Affiliations:** 1 Elderly Care Medicine, East Surrey Hospital, Redhill, GBR; 2 General Surgery, Princess of Wales Hospital, Cwm Taf Morgannwg University Health Board, Bridgend, GBR; 3 Urology, KIMS Al Shifa Super Speciality Hospital, Perintalmanna, IND; 4 Internal Medicine, University of Bahri, Khartoum, SDN; 5 General Physician, University of Perpetual Help System, Hyderabad, IND; 6 Internal Medicine, City Medical Centre, Kandhkot, PAK; 7 Internal Medicine, Davao Medical School Foundation, Inc., Davao, PHL; 8 Internal Medicine, Chandka Medical College, Larkana, PAK; 9 Internal Medicine, Jeejal Medical Center, Larkana, PAK; 10 Medicine, Elizabethtown College, Islamabad, PAK

**Keywords:** acetate, butyrate, fecal scfas, insulin resistance, metabolic syndrome, obesity, propionate

## Abstract

Fecal short-chain fatty acids (SCFAs), such as acetate, propionate, and butyrate, are gut microbial metabolites that influence energy balance, glucose regulation, lipid metabolism, and inflammation. Altered SCFA production has been associated with metabolic disorders, including obesity and type 2 diabetes, but its role remains unclear. This systematic review synthesized evidence from human studies assessing fecal SCFA concentrations and metabolic outcomes. A literature search of PubMed/MEDLINE, Embase, Scopus, and the Cochrane Library (up to June 2025) was conducted following Preferred Reporting Items for Systematic Reviews and Meta-Analyses (PRISMA) 2020 guidelines. Of 94 records identified, seven studies met the inclusion criteria, including cross-sectional and interventional designs. A total of 1650 participants, excluding meta-analysis, have been compared. Elevated fecal SCFA levels were frequently associated with increased adiposity, inflammation, and gut microbiota dysbiosis, while targeted SCFA supplementation, particularly with propionate or butyrate, improved insulin sensitivity and reduced energy intake. Risk of bias was generally moderate due to small sample sizes and variability in SCFA measurement methods. Overall, fecal SCFAs appear to act as both biomarkers and modulators of metabolic health. Larger, standardized longitudinal studies are required to clarify their causal role and therapeutic potential in obesity, metabolic syndrome, and related conditions.

## Introduction and background

Short-chain fatty acids (SCFAs), principally acetate, propionate, and butyrate, are primary metabolites produced by the colonic fermentation of dietary fibers by gut microbiota. These metabolites perform critical physiological functions, serving as energy substrates for colonocytes and acting as signaling molecules that influence host metabolic and immune processes. Mechanistically, SCFAs activate G-protein-coupled receptors (FFAR2 and FFAR3), modulate enteroendocrine hormones such as glucagon-like peptide-1 (GLP-1) and peptide YY (PYY), and inhibit histone deacetylases, thereby impacting energy homeostasis, appetite regulation, and systemic inflammation [1]. Through these pathways, SCFAs provide a mechanistic link between diet, the gut microbiome, and host metabolic health. Metabolic disorders, including obesity, type 2 diabetes mellitus (T2DM), and metabolic syndrome, are among the most pressing global health challenges, with obesity affecting over 650 million adults and T2DM projected to impact more than 643 million individuals worldwide by 2030 [2]. These conditions significantly increase the risk of cardiovascular disease, organ dysfunction, and mortality, creating an urgent need for novel biomarkers and therapeutic strategies.

The gut microbiota has emerged as a key determinant of metabolic health, and SCFAs are increasingly recognized as pivotal mediators within this gut-metabolic axis [3]. Despite evidence supporting SCFAs’ beneficial effects, such as enhanced insulin sensitivity, improved glucose homeostasis, and reduced systemic inflammation, conflicting data exist. Interventional studies have demonstrated that targeted SCFA supplementation, particularly propionate and butyrate, can improve metabolic outcomes and appetite regulation. Conversely, several observational studies associate elevated fecal SCFA concentrations with obesity, gut dysbiosis, and heightened inflammatory responses [4]. These discrepancies suggest that fecal SCFA levels may reflect complex interactions between microbial production, intestinal absorption, dietary patterns, and host physiology. Whether high fecal SCFA concentrations signify a protective mechanism, a consequence of impaired absorption, or a marker of dysbiosis remains unresolved. Given these uncertainties, systematic synthesis of available human evidence is essential to clarify the relationship between fecal SCFAs and metabolic disorders. Understanding these associations is critical for determining whether fecal SCFAs can serve as reliable biomarkers or therapeutic targets for metabolic health. This systematic review aims to evaluate and synthesize evidence from human studies examining the associations between fecal SCFA concentrations and metabolic disorders, including obesity, T2DM, and metabolic syndrome, while identifying mechanistic insights, clinical implications, and research gaps to inform future interventions.

## Review

Materials and methods

Search Strategy

This systematic review adhered to the Preferred Reporting Items for Systematic Reviews and Meta-Analyses (PRISMA) 2020 guidelines to ensure transparency [5]. A comprehensive literature search was conducted in PubMed/MEDLINE, Embase, Scopus, and the Cochrane Library. We used combinations of Medical Subject Headings (MeSH) terms and keywords such as fecal SCFAs, acetate, propionate, butyrate, metabolic syndrome, type 2 diabetes, obesity, insulin resistance, and cardiometabolic risk. Boolean operators AND/OR optimized sensitivity and specificity. Searches were limited to human studies published in English up to June 2025.

Eligibility Criteria

Studies were selected using the PICO (Population, Intervention/Exposure, Comparator, Outcome) framework to ensure alignment with the review objectives [6]. This review focuses on adults or adolescents with metabolic disorders (Population, P), including obesity, type 2 diabetes, and metabolic syndrome. The primary intervention/exposure (I) is the measurement of fecal SCFA levels, compared with healthy individuals or no-disease controls (C). The outcomes (O) of interest include associations between fecal SCFA concentrations and metabolic parameters such as adiposity, insulin resistance, lipid profiles, hypertension, glucose regulation, and markers of inflammation. Studies were eligible if they involved human participants, measured fecal SCFA concentrations, reported defined metabolic outcomes (e.g., BMI, lipid profiles, glucose levels, or blood pressure), included a comparison/control group or correlation analysis, and were published in peer-reviewed journals in English. Studies were excluded if they were animal or in vitro studies, editorials, reviews, conference abstracts, or lacked defined metabolic outcomes.

Study Selection

Two reviewers (blinded to each other’s decisions during the initial phase) independently screened all titles and abstracts identified through the database searches to assess relevance based on predefined inclusion and exclusion criteria. Articles deemed potentially eligible were retrieved for full-text review. Any discrepancies at this stage were resolved through consensus discussions, and if disagreement persisted, a third senior reviewer adjudicated the decision. Studies were excluded if they were animal or in vitro experiments, reviews, conference abstracts, editorials, or lacked defined metabolic outcomes related to SCFAs. The selection process was documented using a PRISMA 2020-compliant flow diagram, including the number of records identified, screened, excluded, and finally included, with reasons for exclusion at the full-text stage.

Data Extraction

Data extraction was carried out independently by two reviewers using a standardized and pre-tested form to ensure accuracy and consistency. Extracted information included author, year of publication, study design, sample size, population characteristics, intervention or exposure details such as SCFA measurement method and concentrations (acetate, propionate, butyrate), comparators, and metabolic outcomes including BMI, insulin resistance, glycemic control, lipid profiles, and inflammatory markers. Additional variables, such as gut microbiota composition and gut barrier integrity, were recorded where reported. Key findings, statistical significance, and effect estimates were noted. All extracted data were cross-verified by both reviewers to minimize errors.

Risk of Bias Assessment

Risk of bias was assessed independently by two reviewers using validated tools. Observational studies were evaluated using the Newcastle-Ottawa Scale (NOS), which examines selection, comparability, and outcome domains, with scores of 7-9 considered low risk, 4-6 moderate, and ≤3 high risk of bias [7]. Randomized controlled trials were assessed using the Cochrane Risk of Bias tool version 2 (RoB 2), which evaluates bias across domains, such as randomization, deviations from intended interventions, missing data, outcome measurement, and selective reporting [8]. Disagreements in quality assessment were resolved through discussion, and overall judgments were summarized in a risk-of-bias table.

Data Synthesis

Due to substantial heterogeneity in study designs, populations, SCFA measurement techniques, and reported outcomes, a meta-analysis was not feasible. Instead, a narrative synthesis was conducted to thematically organize findings into key domains: associations between fecal SCFA concentrations and adiposity measures such as BMI and body fat distribution; correlations between SCFA levels and metabolic parameters, including insulin resistance, glucose regulation, and lipid profiles; and relationships between SCFA concentrations and inflammatory markers or gut barrier integrity. Where applicable, comparisons between observational studies and interventional trials were highlighted to differentiate the effects of endogenous SCFA production from targeted supplementation. Study characteristics contributing to heterogeneity, including population demographics, dietary patterns, and analytical methods, were considered during synthesis. Although a sensitivity analysis based on study quality was planned, it could not be performed due to the small number of included studies.

Results

Study Selection Process

**Figure 1:**
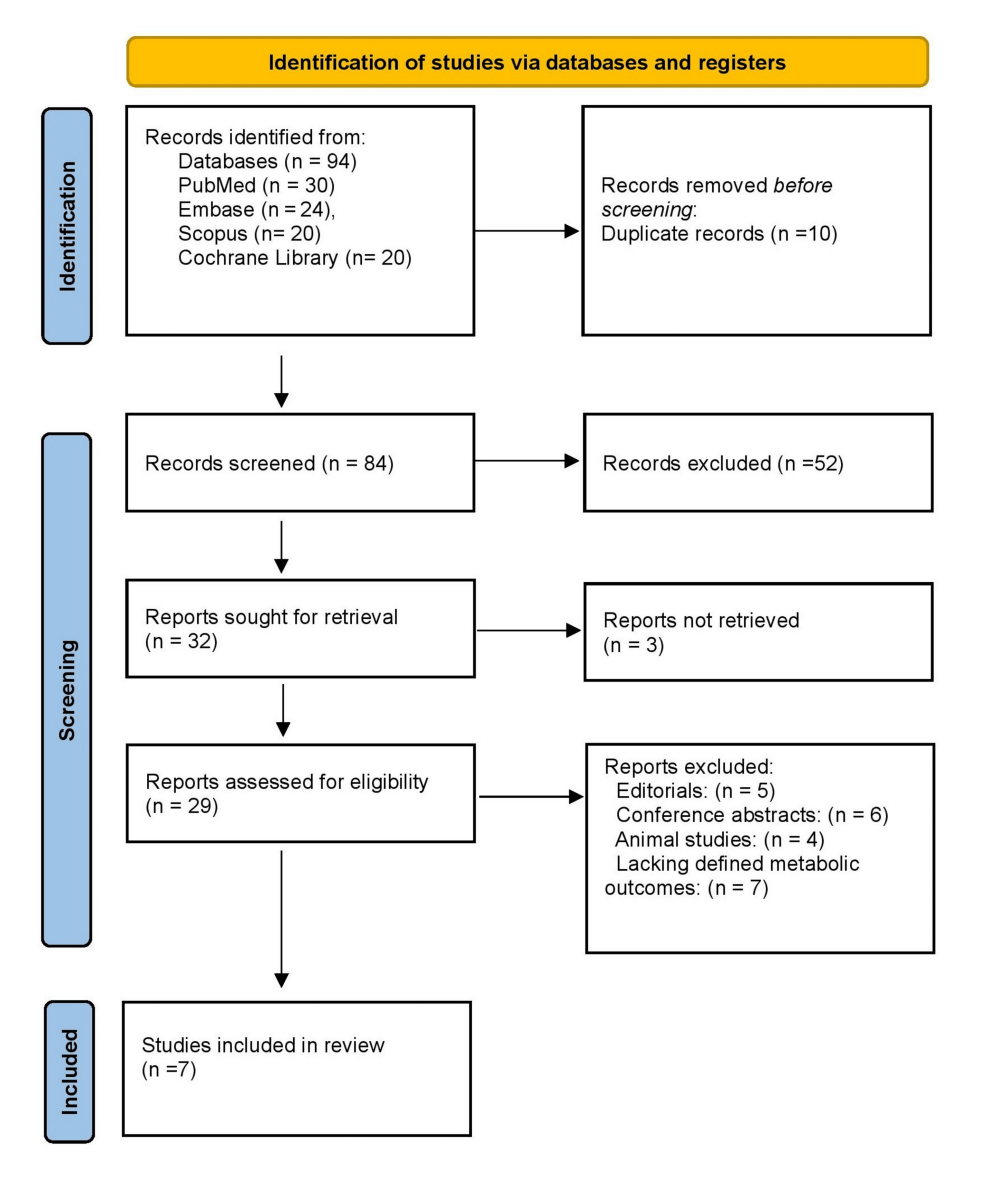
The Preferred Reporting Items for Systematic Reviews and Meta-Analyses (PRISMA) flowchart represents the study selection process

As illustrated in Figure 1, a total of 94 records were identified through database searches, including PubMed/MEDLINE (n = 30), Embase (n = 24), Scopus (n = 20), and the Cochrane Library (n = 20). After removing 10 duplicates, 84 records were screened, of which 52 were excluded based on titles and abstracts. Following full-text assessment of 32 reports, 25 were excluded for reasons such as being case reports, editorials, conference abstracts, animal studies, or lacking defined outcomes. Ultimately, seven studies were included in the final review.

Characteristics of the Selected Studies

As shown in Table 1, the seven studies differed in design, population, and SCFA measurement methods, including gas chromatography-mass spectroscopy (GC-MS) and high-performance liquid chromatography (HPLC). Most were cross-sectional, with two interventional trials. Populations ranged from community adults to women with gestational diabetes and patients with metabolic syndrome. Comparators were typically healthy individuals, and outcomes included BMI, insulin resistance, lipid profiles, and inflammation. Elevated fecal SCFAs were frequently linked to gut dysbiosis, adiposity, and inflammatory responses. Anatomical effects were largely unreported, though some studies noted altered gut barrier integrity. Renal outcomes were not directly assessed.

**Table 1:** The characteristics of selected studies BMI - body mass index; GDM - gestational diabetes mellitus; GC-MS - gas chromatography-mass spectrometry; GLP-1 - glucagon-like peptide 1; HbA1c - hemoglobin A1c (glycated hemoglobin); HPLC - high-performance liquid chromatography; hs-CRP - high-sensitivity C-reactive protein; IL-6 - interleukin-6; MetS - metabolic syndrome; PICO - Population, Intervention/Exposure, Comparator, Outcome; PYY - peptide YY; SCFAs - short-chain fatty acids

Authors and Year	Population (P)	Exposure/Condition (I)	Comparator (C)	Outcomes (O)	Pathophysiological Findings	Anatomical Impact	Renal Outcomes
de la Cuesta-Zuluaga et al., 2018 [9]	441 community adults	Elevated fecal SCFA levels	Healthy-weight individuals	BMI, hypertension, gut dysbiosis	Higher fecal SCFAs linked to metabolic risk factors	Reduced gut microbial diversity	Not reported
Yamamura et al., 2021 [10]	568 Japanese adults	High quartile fecal SCFAs	Lower quartiles	Obesity prevalence	SCFAs associated with higher adiposity and inflammation	Altered gut barrier function	Not reported
Roshanravan et al., 2019 [11]	60 metabolic syndrome patients	Oral butyrate supplementation	Placebo	Lipids, HbA1c, BMI	Mixed effects on lipid and glucose regulation	No anatomical data	Not reported
Chambers et al., 2015 [12]	60 healthy and MetS adults	Propionate supplementation	Placebo	Energy intake, GLP-1, PYY	Improved insulin sensitivity and appetite control	No anatomical data	Not reported
Rad et al., 2023 [13]	32 obese women	Low-carb diet increasing SCFAs	Baseline values	Insulin, IL-6, hs-CRP	Higher SCFAs correlated with inflammation	No anatomical data	Not reported
Molan et al., 2024 [14]	45 women with GDM	Elevated fecal SCFAs	Non-GDM pregnant women	Weight gain, insulin resistance	High SCFAs linked with excessive gestational weight gain	Altered microbiota	Not reported
Kim et al., 2019 [15]	246 obese cases and 198 normal controls	Fecal SCFA levels in obesity	Lean controls	Acetate, propionate, butyrate levels	Obese individuals had significantly higher SCFAs	No anatomical data	Not reported

Risk of Bias Assessment

As illustrated in Table 2, the risk of bias assessment reveals that most studies were of moderate quality, mainly due to confounding factors, small sample sizes, and cross-sectional designs that limit causal inference. Observational studies (NOS) were generally well-structured but lacked consistent control for diet and lifestyle. Interventional trials (Cochrane RoB 2) showed lower bias overall, though some had incomplete blinding or unclear randomization. The meta-analysis by Kim et al. [15] was not assessed as it is a secondary source. Overall, the evidence is moderate, highlighting the need for larger, well-controlled studies.

**Table 2:** The risk of bias assessment NOS - Newcastle-Ottawa Scale; RCT - randomized controlled trial; RoB 2 - Cochrane Risk of Bias 2 Tool; SCFA - short-chain fatty acid

Study	Study Design	Risk of Bias Tool	Risk of Bias Rating	Justification
de la Cuesta-Zuluaga et al., 2018 [9]	Cross-sectional observational	Newcastle-Ottawa Scale (NOS)	Moderate	Well-defined population and outcomes; potential confounding from dietary factors not fully controlled.
Yamamura et al., 2021 [10]	Cross-sectional observational	NOS	Moderate	Large sample size but limited adjustment for lifestyle variables.
Roshanravan et al., 2019 [11]	Randomized controlled trial	Cochrane RoB 2	Low to moderate	Randomization described, but blinding and allocation concealment were unclear.
Chambers et al., 2015 [12]	Randomized controlled trial	Cochrane RoB 2	Low	Strong methodology with adequate blinding and outcome assessment.
Rad et al., 2023 [13]	Randomized feeding trial	Cochrane RoB 2	Moderate	Small sample size and incomplete reporting of randomization procedures.
Molan et al., 2024 [14]	Cross-sectional observational	NOS	Moderate	Clear exposure and outcomes, but cross-sectional design limits causal inference.
Kim et al., 2019 [15]	Systematic review and meta-analysis	Not applicable (secondary source)	Not assessed	As this is a meta-analysis, risk of bias is not evaluated with NOS or RoB 2 in this review context.

Discussion

The present systematic review highlights the complex and often paradoxical role of fecal SCFAs in metabolic health. Across the included studies, elevated fecal SCFA concentrations were commonly associated with obesity, metabolic syndrome, and systemic inflammation, while targeted supplementation of SCFAs, particularly propionate and butyrate, demonstrated beneficial metabolic effects such as improved insulin sensitivity and reduced appetite [16]. These findings underscore the dualistic nature of SCFAs as both potential biomarkers of dysregulated metabolism and modulators of therapeutic benefit. The observed positive associations between high fecal SCFA levels and adverse metabolic profiles in cross-sectional studies may reflect underlying gut dysbiosis and altered SCFA handling rather than a direct pathogenic effect. Elevated fecal SCFAs could indicate increased microbial fermentation coupled with impaired colonic absorption or accelerated intestinal transit. In contrast, intervention studies delivering SCFAs directly to the colon suggest that controlled supplementation improves glucose homeostasis and reduces energy intake through gut hormone modulation and enhanced satiety signaling. This divergence supports the hypothesis that the metabolic impact of SCFAs depends on their site of action and systemic availability rather than absolute fecal concentrations [17].

Mechanistically, SCFAs influence host metabolism through several pathways. Activation of G-protein-coupled receptors (FFAR2, FFAR3) on enteroendocrine and immune cells regulates secretion of GLP-1 and PYY, improving insulin sensitivity and appetite control. Additionally, SCFAs act as inhibitors of histone deacetylases, exerting anti-inflammatory and epigenetic effects. However, excessive SCFA production in the context of an energy-rich diet and dysbiotic microbiota may amplify caloric extraction and promote adiposity, partially explaining the association between high fecal SCFA levels and obesity. These mechanisms indicate that SCFA effects are context-dependent, modulated by diet composition, microbiota diversity, and host absorption efficiency. Notably, Westernized diets high in fat and low in fiber may skew microbial metabolism toward SCFA patterns that exacerbate metabolic risk. Conversely, diets rich in prebiotic fibers promote beneficial butyrate-producing bacteria that enhance gut barrier function and reduce inflammation [18].

From a clinical perspective, fecal SCFAs hold promise as non-invasive biomarkers of gut microbial activity and metabolic risk. However, their diagnostic utility is limited by heterogeneity in measurement methods, lack of standardized cut-off values, and confounding factors such as diet, medication use, and bowel habits. Therapeutically, strategies to modulate SCFA production via dietary fiber supplementation, prebiotics, probiotics, or direct SCFA delivery represent an emerging approach in metabolic disease management. Propionate and butyrate supplementation, for instance, have demonstrated potential in reducing appetite and improving glycemic control, suggesting a role for targeted interventions rather than generalized SCFA elevation. Previous reviews and meta-analyses similarly report inconsistent associations between fecal SCFAs and obesity, reinforcing the complexity of interpreting fecal measurements as proxies for systemic exposure. Kim et al. [15] found that individuals with obesity exhibited higher fecal SCFA levels than lean controls, but causality remains uncertain due to the predominance of cross-sectional designs. Recent studies emphasize the need to distinguish between luminal and circulating SCFAs, as plasma SCFAs reflecting absorbed fractions may correlate more strongly with metabolic outcomes than fecal concentrations.

The findings of this review should be interpreted in light of several limitations. Most included studies were observational and cross-sectional, restricting causal inference. Sample sizes were generally small, and heterogeneity in analytical techniques (GC-MS, HPLC) complicates comparisons across studies. Moreover, dietary intake and lifestyle factors were variably controlled, introducing potential confounding. Future research should prioritize longitudinal studies with standardized SCFA measurement protocols, integration of dietary assessment, and concurrent analysis of fecal and plasma SCFA profiles. Additionally, mechanistic trials assessing dose-response relationships and long-term clinical outcomes of SCFA supplementation are warranted [19]. Emerging technologies, such as metabolomics and microbiome sequencing, offer opportunities to refine our understanding of SCFA dynamics within the gut ecosystem. Personalized interventions based on microbiota composition and metabolic phenotype may optimize SCFA-mediated benefits while minimizing adverse effects. Furthermore, exploring the interaction of SCFAs with bile acids, gut peptides, and inflammatory mediators could provide insights into their broader metabolic impact. Overall, fecal SCFAs represent a promising but complex biomarker of metabolic health, reflecting both microbial activity and host absorption dynamics. While supplementation strategies targeting SCFA pathways hold therapeutic potential, the variability in current evidence underscores the need for standardized methodologies and well-controlled clinical trials to elucidate causality and clinical applicability.

## Conclusions

Fecal SCFAs, including acetate, propionate, and butyrate, are key microbial metabolites that link gut microbiota to host metabolism, exerting both beneficial and detrimental effects depending on the physiological context. While targeted SCFA supplementation has shown promise in improving insulin sensitivity and appetite regulation, elevated fecal SCFA levels have also been associated with increased adiposity, inflammation, and insulin resistance. This paradox highlights the complexity of SCFA action and the influence of host and environmental factors. Current evidence is limited by predominantly cross-sectional designs, small sample sizes, and inconsistent SCFA quantification methods. Future research should prioritize large-scale, longitudinal studies with standardized analytical techniques and integrate multi-omics approaches to better define SCFA-host interactions. Stratified analyses based on age, BMI, and microbiota profiles may further clarify individual responses to SCFAs. Such efforts are essential to evaluate the therapeutic potential of SCFAs in metabolic disorders and inform precision nutrition strategies.
